# Continental Refugium in the Mongolian Plateau during Quaternary Glacial Oscillations: Phylogeography and Niche Modelling of the Endemic Desert Hamster, *Phodopus roborovskii*

**DOI:** 10.1371/journal.pone.0148182

**Published:** 2016-02-03

**Authors:** Xue Lv, Lin Xia, Deyan Ge, Zhixin Wen, Yanhua Qu, Liang Lu, Qisen Yang

**Affiliations:** 1 Key Laboratory of Zoological Systematics and Evolution, Institute of Zoology, Chinese Academy of Sciences, Beijing 100101, China; 2 College of Life Science, University of Chinese Academy of Science, Beijing 100049, China; 3 National Institute for Communicable Disease Control and Prevention, Chinese Centre of Disease Control and Prevention, Beijing 102206, China; Sichuan University, CHINA

## Abstract

The Mongolian Plateau (MP), which is situated in the interior of Asia and possesses a typical continental climate, experienced harsh climatic conditions during the Quaternary glacial fluctuations. Although these events likely had huge impacts on the local animal populations, the current effects have hardly been explored. To investigate whether the MP supported a refugium along an oceanic-continental gradient (ROCG), and whether this refugium was glacial or interglacial, we investigated the demographic and phylogeographic history of an endemic mammal species, the desert hamster *Phodopus roborovskii*. We reconstructed the demographic variation, the phylogeographic diffusion, and modelled the potential habitat during historical periods. The genetic diversity in the MP was the highest among all the localities, and the MP was a suitable habitat throughout the modelled historical periods. A phylogeographic diffusion analysis emphasized the importance of the MP as the centre of origin, preservation and spread for *P*. *roborovskii*. The homogeneous landscape provided the opportunity for a wide gene flow, which resulted in low resolution of the phylogenetic relationships. Moreover, *P*. *roborovskii* was favoured by the interglacial condition, with both its demographical and geographical ranges expanded within the interglacial periods. The range variation from the Last Glacial Maximum to the current condition reflects a distinct longitudinal shift, while both ranges largely contracted from that of the Last Interglacial. Our results support that the MP served as a refugium and spread centre for *P*. *roborovskii* during the Quaternary climate fluctuations. The interglacial expansion and the longitudinal shifts highlighted the important effects of precipitations on the distribution range of species adapted to arid and semi-arid during glacial oscillations.

## Introduction

The role of the Pleistocene climatic oscillations in shaping species distributions, genetic diversification and demography has been widely studied [1]. Particularly, the pattern of elevational and latitudinal range shifts during glacial cycles remains the focus of scientific debate (e.g. [2, 3]). Recently, the concept of longitudinal range shifts along an oceanic-continental gradient (ROCG) has been introduced [4]. This gradient of oceanic–continental climate was also significantly variable during the Pleistocene glacial cycles. Species with “oceanic” adaptations are suited to more humid environments with less seasonal variability, whereas species with “continental” adaptations have the opposite characteristics and are adapted to low precipitations and arid environments with distinct seasonal temperature variability. Interglacial refugia may therefore, be needed for continent-adapted species according to these features [5]. A previous study on a continent-adapted species, the European ground squirrel (*Spermophilus citellus*) in the south-eastern European steppe [5], showed a demographical increment due to the range expansion of the steppe environment during the glacial periods. However, range fluctuation trends can only be speculated by demographic dynamics. Further comprehensive studies are required to better understand the geographical evolutionary processes that affect continent-adapted taxa.

The Mongolian Plateau (MP) is situated in the interior of Asia and possesses a typical continental climate. This region contains the entire territory of Mongolia and the Inner Mongolian Autonomous Region of China. It extends from Lake Baikal, in the north, to the eastern edge of the Qinghai-Tibet Plateau (QTP), in the south [6]. As the centre of the arid and semi-arid biomes of East Asia, the MP harbours a high biodiversity, which contrasts with its low productivity [7]. Currently, more attention [2, 8, 9] has been paid to the phylogeographical history of organisms in mountain areas during the glacial cycles of the Quaternary (e.g., in the QTP and adjacent areas [2, 8–10]). In contrast, arid and semi-arid regions such as the MP have barely been examined (but see [11, 12]). Although large glaciers did not form on the MP, the uplift of the QTP profoundly influenced this region [13, 14], which was further affected by fluctuations in vegetation and rapid community succession. Indeed, the rapid succession of vegetation types from glacial desert to interglacial broadleaved deciduous forests [15] made it difficult for several organisms to survive on the MP during the Quaternary glacial fluctuations. Therefore, the Quaternary glacial cycles had a strong influence on the terrestrial animals due to both climatic fluctuations and vegetation shifts [16]. However, preliminary evidence was found for refugia in the MP for mesobuthid scorpions during the Quaternary climatic fluctuations [17], which suggest that the MP was a potential reserve centre during these climatic changes. Therefore, how did these endemic animals resist and respond to climate fluctuations under such harsh environmental conditions? To answer these questions, it is necessary to explore the evolutionary history and biogeographical patterns of endemic taxa from this region with an extensive sampling.

As one of the endemic species of the MP, the desert hamster (*Phodopus roborovskii*) shows a high tolerance to cold and drought environments with large seasonal climatic variation [18]. This species exclusively inhabits areas with loose sand and sparse vegetation and avoids solid clay substrates and environments covered with dense vegetation that are not suitable for burrowing [19]. These features make the *P*. *roborovskii* an ideal candidate for studying how continent-adapted species responded to the environmental changes during the Quaternary climatic fluctuations in the MP. In a recent study by [20], individuals from the northern part of its range, which includes Mongolia, Tuva and Kazakhstan, were sequenced and analysed. Their results indicated that the Quaternary climate fluctuations had a major impact on the current distribution of this species, whereas the colonization of the northern range probably took place in recent times. However, the available samples of *P*. *roborovskii* in this study are insufficient to cover its current distribution, and samples from China are particularly lacking, which includes both the highest and lowest elevational distribution for this species. Therefore, a general clarification of the phylogeographic history of this species is needed, which will require studies based on a more comprehensive sampling and a wider range of sequence data.

**Fig 1:**
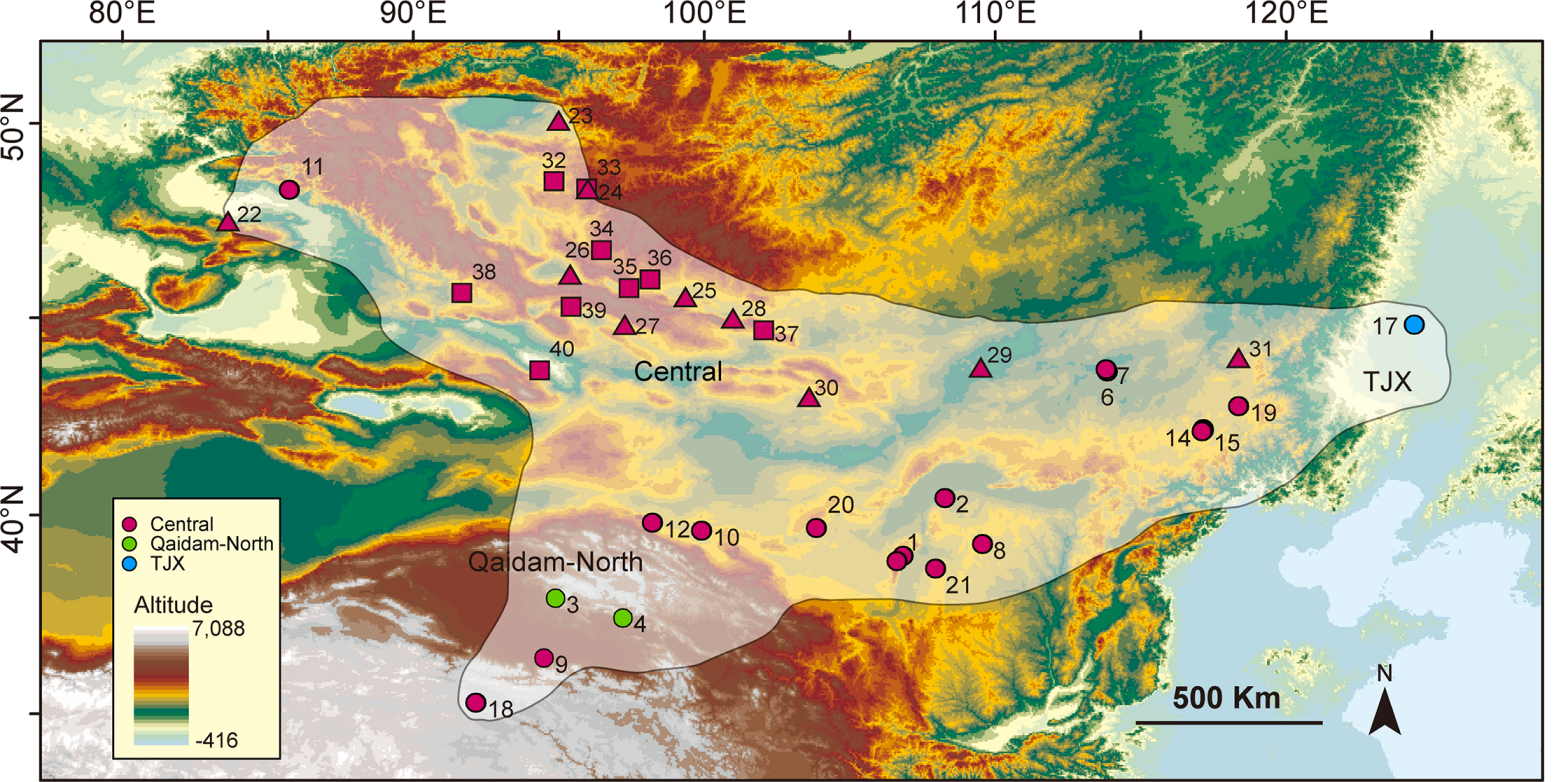
Sampling locations of *P*. *roborovskii*. Round points represent the molecular sampling localities of this study; squares correspond to the sampling sites of [20], and the triangles correspond to those of [21]. All sampling localities are marked with locality number (see S1 Table), and the colour represents population assignment. The territorial altitude is shown with a legend at bottom left of the figure. The extant distribution range is showed in grey.

In this study, we included a large number of sampling localities, together with those from [20], and covered nearly all the contemporary distribution range of *P*. *roborovskii* (Fig 1). Using this dataset, we then asked the following questions: 1) did the MP served as preservation centre for *P*. *roborovskii* during unfavourable periods of climate change? And if it did, 2) did the refugium served for the interglacial period or for the glacial period? 3) did the *P*. *roborovskii* shifted its habitat along the oceanic-continental (OC) axis during the climatic fluctuations? Considering the biological and ecological characteristics of continental climate-dwelling animals, we proposed the following two hypotheses: 1) the MP served as an interglacial refugium and a glacial expansion centre for *P*. *roborovskii*; and 2) *P*. *roborovskii* shifted its range along the OC axis during the climatic fluctuations. To examine these hypotheses, we comprehensively analysed the historical demographic and geographical variation of *P*. *roborovskii*.

## Materials and Methods

### Ethics statement

This study was carried out in strict accordance with animal research protocol IOZ-2006 approved by the Animal Care Committee of Institute of Zoology, Chinese Academy of Sciences (IOZ, CAS). The desert hamsters were captured with permission of the local Protection and Research Centre, and the Forestry Administration of Inner Mongolia, Xinjiang, Gansu, Qinghai, Jilin, Ningxia and Hebei Provinces. All desert hamsters were captured by cage, and sacrificed by decapitation immediately after capture. All efforts were made to minimize potential pain and suffering. All tissue samples were preserved in 95% ethanol upon collection.

### Data collection

A total of 77 samples from 21 localities in China were newly sequenced for the present study. In addition, we downloaded 41 sequences from the samples collected in Mongolia, Kazakhstan and Russia. These sequences included one Cytochrome b gene (Cytb) from [22] (GenBank#AJ973391), one from [23] (EF025539), 26 from Meshchersky [20] (GU797444-GU797469), 13 from [21] (JQ771080-JQ771092), and 26 D-Loop sequences from [20] (GU812865-GU812890), which were also used in the analyses (Fig 1, see S1 Table).

Two mitochondrial DNA (mtDNA) genes were amplified for this study: Cytb and D-Loop. Information on the primers and the major references is the following: Cytb, L14728 and H15985 [24]; D-Loop, L16007 and H00651 [25]. Polymerase chain reaction (PCR) amplifications were performed in a total reaction volume of 25 μl, which contained 3 μl template DNA (~60 ng μl^-1^), 1 μl primers (10 mM), 2 μl dNTPs (2.5 mM), 2.5 μl 10× PCR buffer (that contained 15 mM MgCl_2_), and 0.25 μl Taq polymerase (Takara Shuzo Co. Ltd., Otsu, Japan). The reaction were adjusted to a final volume of 25 μl with ddH_2_O. The following amplification conditions were used for all the markers: 94°C for 10 min, followed by 35 cycles of 94°C for 1 min, 54°C for 45 s, and 72°C for 90 s, followed by a final extension step at 72°C for 10 min.

The complete sequences were assembled using Seqman II and Editseq (DNASTAR, Madison, WI, USA), aligned using Clustal W [26] implemented in MEGA5 [27] and, finally, combined in MacClade4 [28].

### Phylogenetic and population genetic analyses

First, the best fit model of nucleotide substitution was selected using the Akaike information criterion (AIC) in jModelTest v2.1.4 [29], with a partition of three codons of Cytb and a partition between loci (Cytb and D-Loop).

Two methods were used for the phylogenetic inference. First, we produced median-joining [30] networks for the concatenated mtDNA sequences in POPART [31]. Second, a phylogenetic Bayesian tree based on concatenated mtDNA was constructed in MrBayes 3.2 [32]. The MrBayes analysis was ran for a total of 10,000,000 generations and was sampled every 1,000 steps, with a final burn-in value of 100,000.

**Fig 2:**
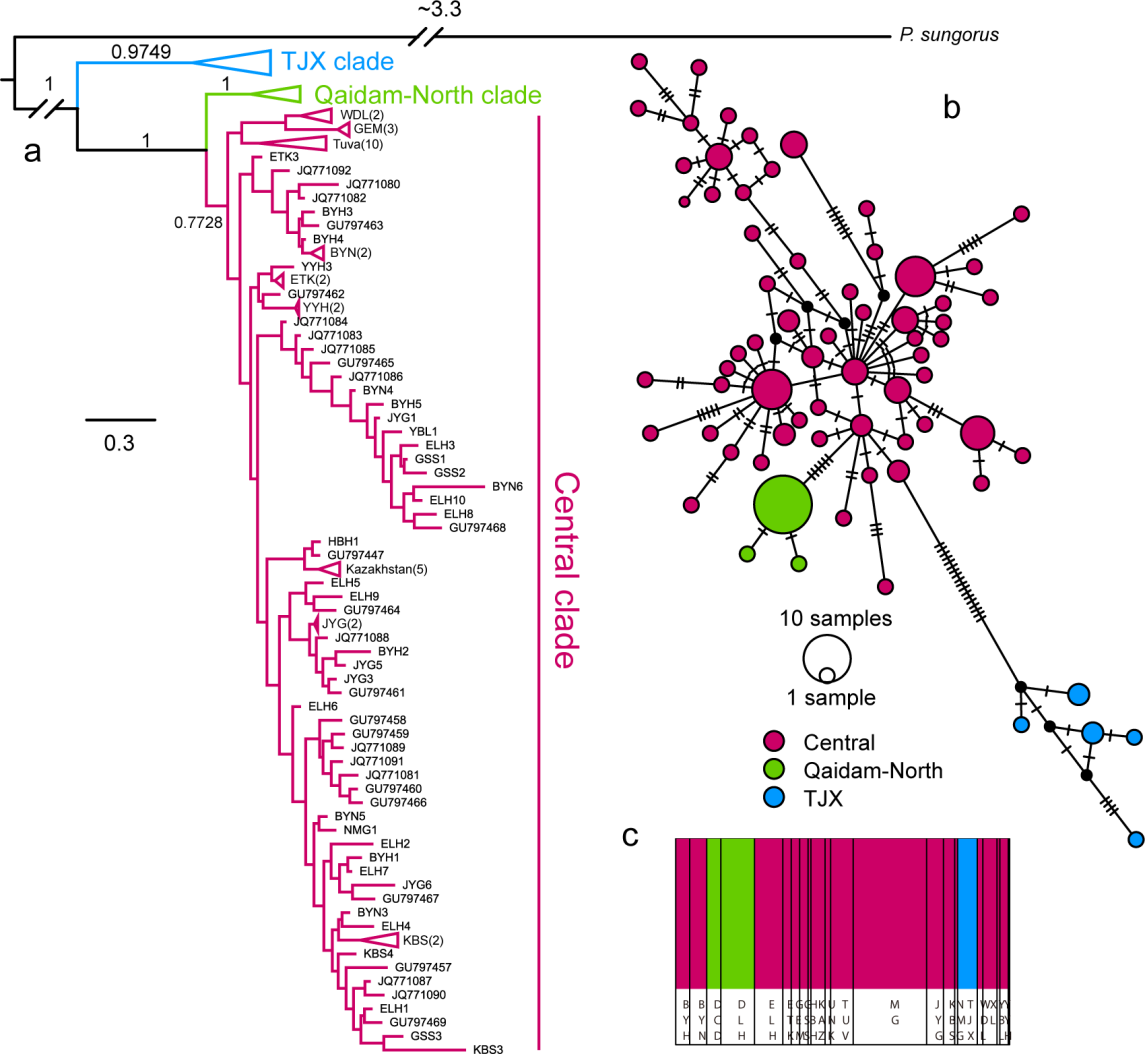
Results from the Bayesian phylogenetic analysis, the MJ network and the population cluster from BAPS. The phylogenetic tree was collapsed to improve clarity.

To further subdivide the population, we implemented two analyses. First, we used a Bayesian cluster method in BAPS [33] to detect the population genetic structure. We conducted a mixture analysis (cluster of linked loci), with 10 replicates on each number of genetic groups. The maximal group numbers were set to 2–6. Based on the results of BAPS, we further examined the population delimitation in the software BP&P [34]. The reversible-jump Markov chain Monte Carlo (rjMCMC) analyses were ran for 100,000 generations with a burn-in phase of 8,000. We used a gamma prior G (2, 1,000) on the population size parameters (θs), and a gamma prior G (2, 2,000) on the age of the root in the species tree (τ0). The other parameters were assigned to be the prior default. Each analysis was run twice with different starting seeds to ensure the consistency. Because the species delimitation for three species is highly supported (see results) and integrated with the results of phylogenetic analyses, we subdivided all the individuals into three clades: the Central clade, the Qaidam North (QN) clade and the TJX clade (Fig 2) in the following analyses.

To analyse the general patterns of diversity in the mtDNA data, the nucleotide diversity (π), the mean number of pairwise nucleotide differences (k), the number of haplotypes (nh) and the haplotype diversity (h) were calculated for each clade, which was performed using DnaSP [35]. The significance was tested using 10,000 permutations in Arlequin [36].

To further visualize the geographical pattern of genetic diversity, the nucleotide diversity (π) was spatially interpolated according to [37], with the application of three methods: the Kriging method, the inverse distance weighted (IDW) method and the natural neighbour method, all implemented in the ‘Geostatistical Analyst’ of ArcGIS 10.0 (ESRI, Redlands, CA, USA). The genetic diversity was recalculated by resampling the localities contained in a buffer of 1° ratio around each sample locality to include at least two individuals per locality in each calculation. Hence, we sampled a total of 24 localities.

### Demographic analyses

The historical demography of *P*. *roborovskii* was assessed using three methods. First, Fu’s F [38], Tajima’s D and R2 [39] statistics were calculated for each locality and each clade. These statistics can be used to detect population growth under a model of sudden expansion. In total, 10,000 coalescent simulations of a neutral evolving model were generated in Arlequin. The significance of the R2 statistic was calculated in DnaSP with 10,000 coalescent simulations. Furthermore, we tested the pairwise mismatch distribution of the major clades to identify the signatures of the demographic expansion. This method was implemented for concatenated mtDNA in DnaSP.

To explore the population fluctuations over time, we used extended Bayesian skyline plots (EBSP) [40, 41] implemented in BEAST [42] to estimate the posterior distribution of the effective population size. Substitution models were used as in the Bayesian phylogenetic analysis. The molecular clock model was tested in PAUP* [43] (rejected, p < 0.001) and was set as an uncorrelated lognormal relaxed clock. We used two mutation rates of 1.5 and 6% per million years for the mtDNA of rodents [3, 44] as upper and lower bounds, respectively. The analyses were run for 50,000,000 steps and sampled every 5,000 steps. The MCMC convergence was assessed in TRACER [45] and the effective sample size (ESS) values for all the parameters exceeded 200. Each analysis was run twice to ensure the congruence between results.

### Geographical analysis

The range variation of *P*. *roborovskii* was assessed with two methods. First, to examine the phylogeographic diffusion pattern of *P*. *roborovskii* based on molecular and extant distribution data, we performed continuous phylogeographic diffusion analyses in BEAST v 2.3.1 [46]. To obtain a better convergence, we subsampled the data set to include one individual per haplotype per locality, with resulted in a total of 81 individuals. Other settings of priors were the same as those of the EBSP analyses. The analysis was run for 30,000,000 generations with sampling every 3,000 generations. The final convergence was assessed with ESS. The spatial-temporal diffusion pattern was then reconstructed with Spread [47] and visualized with Google Earth (Google, California, USA, available at http://google.com/earth/).

Second, to examine the consequences of climate change on the range shifts of *P*. *roborovskii*, MaXent 3.3.2 [48, 49] was used to reconstruct the potential habitat of *P*. *roborovskii* during the Quaternary climate fluctuations. We obtained climatic data from three periods: the present, the LGM (~21,000 years BP), and the Last Interglacial (LIG, ~120,000–140,000 years BP) periods. Nineteen bioclimatic variables were downloaded from the WorldClim database [50] (available at http://www.worldclim.org/) at a resolution of 2.5 arc-minutes. The variables were masked to include only 20° to 70°N and 60° to 150°E. A total of 75 sets of species occurrence data were collected, which included sampling localities, museum records (provided by the National Zoological Museum of China, NZMC), and occurrence records from the GBIF database (available at http://www.gbif.org/). First, we tested the correlation among climatic variables and chose only one variable if two variables were highly correlated (Pearson’s coefficiency > 0.8) to eliminate the effect of over-fitting. Second, we tested the model fitness by examining beta regulation values from 1 to 20. These preliminary processes were performed in ENMtools 1.4.4 [51]. The results led us to choose climatic variables of BIO1, BIO2, BIO3, BIO4, BIO8, BIO12, BIO14, BIO15 and BIO16, with a beta value of three. Then, we randomly selected 80% of the occurrence data to construct the model, and 20% of the data were left to test the accuracy of the model. The model used the default convergence threshold (10^−5^), 2,000 maximum iterations and 10 replications. MaXent tests the model by calculating the average area under the receiver operating characteristic curve (AUC) and the binominal probabilities over ten replicate runs. These two metrics indicate the predictive ability of the model.

## Results

### Phylogenetic and population genetics analyses

Three strongly supported clades were recovered according to the Bayesian methods (Fig 2A) based on total mtDNA (Cytb, 1,103 bp; D-Loop, 866 bp). This outcome is consistent with the result from BAPS (Fig 2C), which is also strongly supported (posterior = 1.0) in the BP&P species delimitation analysis. The TJX clade, which contains individuals from the eastern edge of the distribution range in the Jilin Province was remarkably divergent from the other lineages. The Qaidam-North clade included localities from the north of the Qaidam Basin in Qinghai. Samples from the MP and adjacent areas (e.g., Kazakhstan and Tuva) generally lacked a strong structure, and individuals from the same locality often did not cluster together. This pattern was also supported by the mtDNA haplotype networks (Fig 2B).

**Fig 3:**
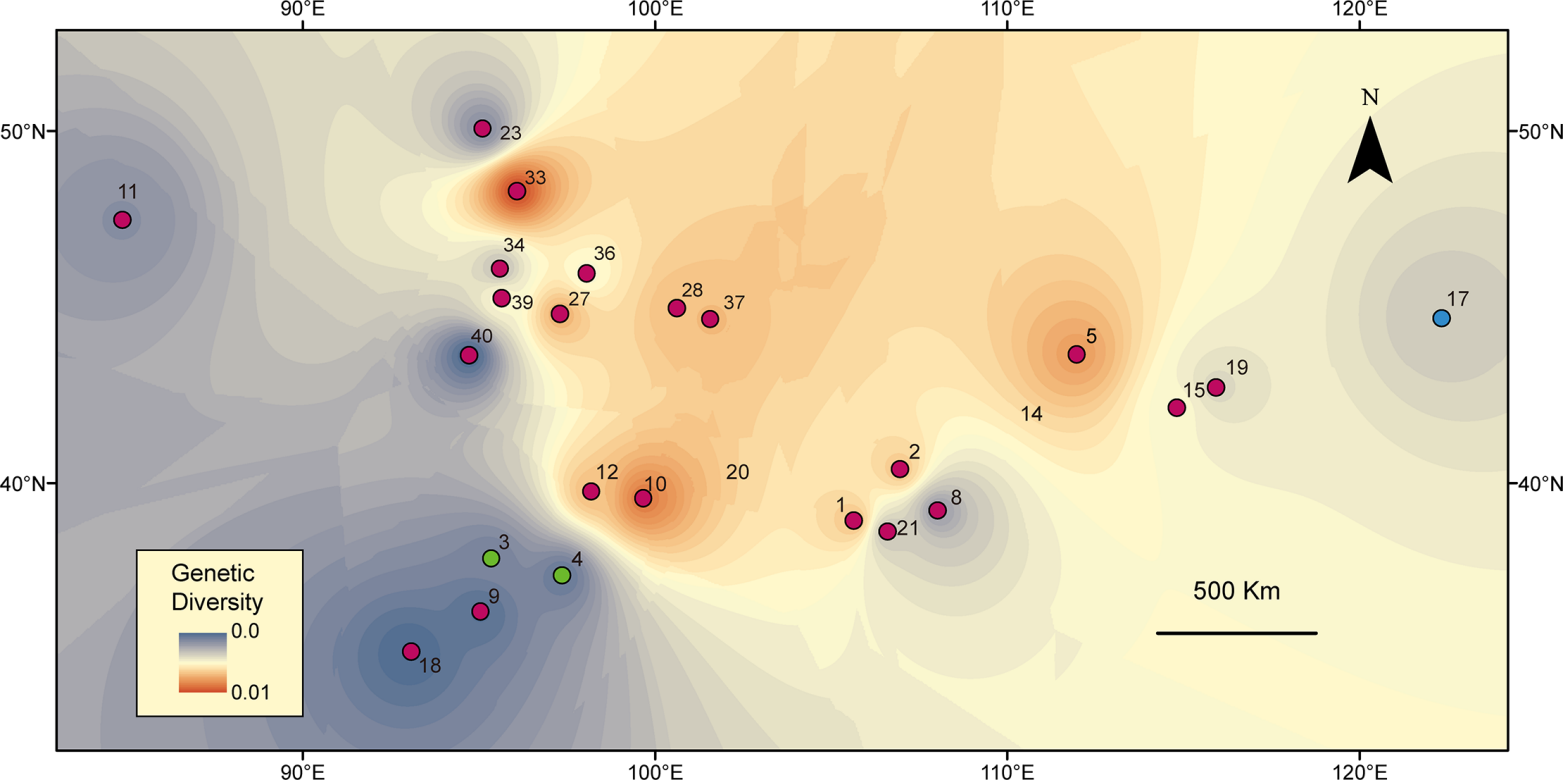
Surface of the interpolated genetic diversity of *P*. *roborovskii*. The red colour indicates a high genetic diversity, and the blue colour indicates a low diversity. The interpolation was conducted under an IDW framework. Numbers represent the sampled localities of Fig 1.

Based on the concatenated mtDNA data, we identified 69 haplotypes from 118 individuals. The nucleotide diversity of the mtDNA was 1.05% and ranged from 0.15% to 0.74% among the clades (Table 1). The geographic patterns of the genetic diversity interpolated with three methods showed congruent results (Fig 3, S1 Fig), and the highest genetic diversity was distributed in the MP.

**Table 1:** Summary statistics and test results of the historical demography based on mtDNA.

Clade	n	nh	π (%)	k	h	Fu's Fs	R2	Tajima's D
**Central**	94	65	0.74	6.76	0.98	-0.36***	0.10***	-1.92**
**TJX**	7	6	0.39	6.90	0.95	0.35	0.16***	-0.04
**Qaidam-North**	17	9	0.15	2.84	0.53	-3.71**	0.14***	-1.2
**All**	118	106	1.05	253.84	0.94	-1.62	0.09***	-1.46

Sampled localities, numbers of individuals (n), number of haplotypes (nh), nucleotide diversity (π), mean number of pairwise nucleotide differences (k), haplotype diversity (h), Fu’s F and Tajima’s D.

**p< 0.01.

***p<0.

### Demographical analyses

**Fig 4:**
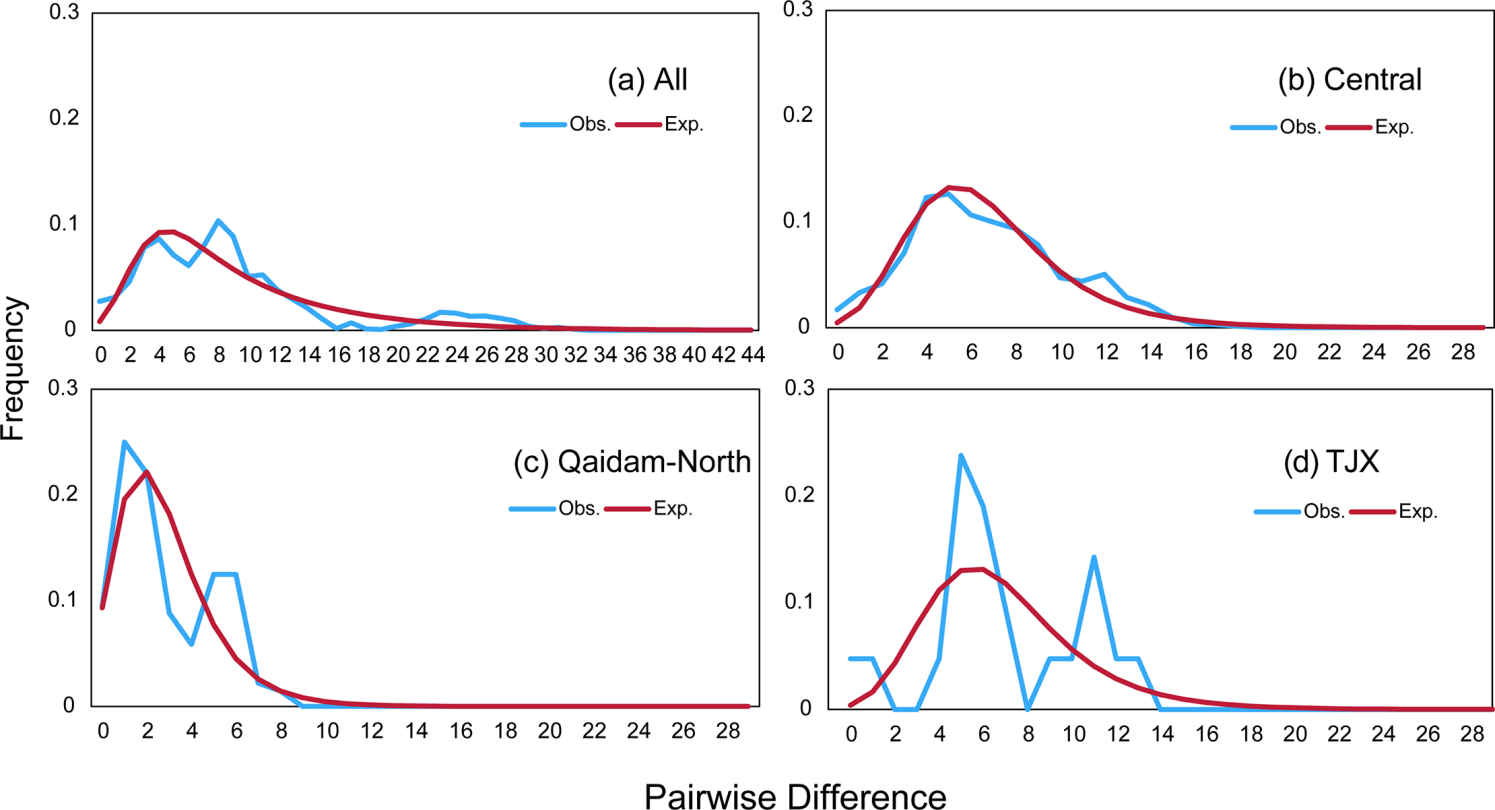
Mismatch distributions of all the main clades. The blue lines indicate the observed frequency of pairwise nucleotide differences between sequence, and the red lines are the expected distribution based on a model of sudden population expansion.

**Fig 5:**
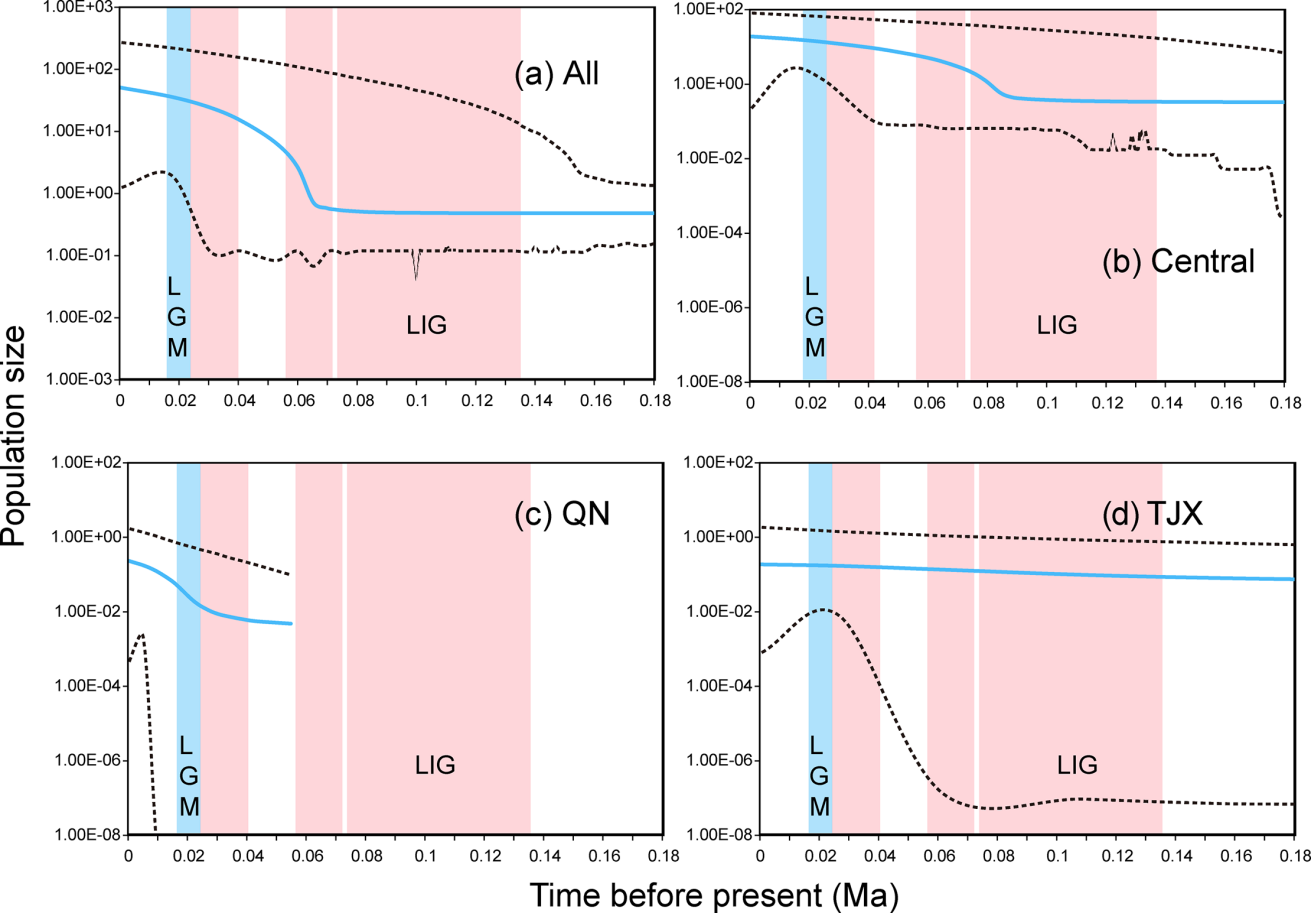
Extended Bayesian Skyline plots for all the main clades of *P*. *roborovskii*. The solid lines indicate the median value of effective population size; the dashed lines denote the 95% highest posterior probability interval. The discarded part showed no change of effective population size. The red-shaded regions represent interglacial periods and were drawn based on the chronology of Quaternary glaciations in China summarized by [52]. The first one represents the Last Interglacial (LIG). The blue shaded-region represents the Last Glacial Maxima (LGM).

For all the individuals, the demographical analyses showed a tendency of population growth. Though not significant in the neutral test (Table 1), a mismatch analysis showed a roughly unimodal distribution (Fig 4A). The results of the EBSP further showed a significant population growth that began at approximately 60 thousands of years ago (Ka, Fig 5A). For the Central clade, the Fu’s Fs and Tajima’s D tests were both significantly negative, as well as the significantly small R2 values (Table 1). Likewise, the results of the pairwise mismatch distributions (Fig 4B) were unimodal, and the results from the EBSP demonstrated that this congruent expansion took place approximately 80 Ka (Fig 5B). The results for the Qaidam-North clade also supported a demographical expansion (except for the multimodal mismatch distribution). Instead, the TJX clade experienced a more stable history.

### Geographical analyses

**Fig 6:**
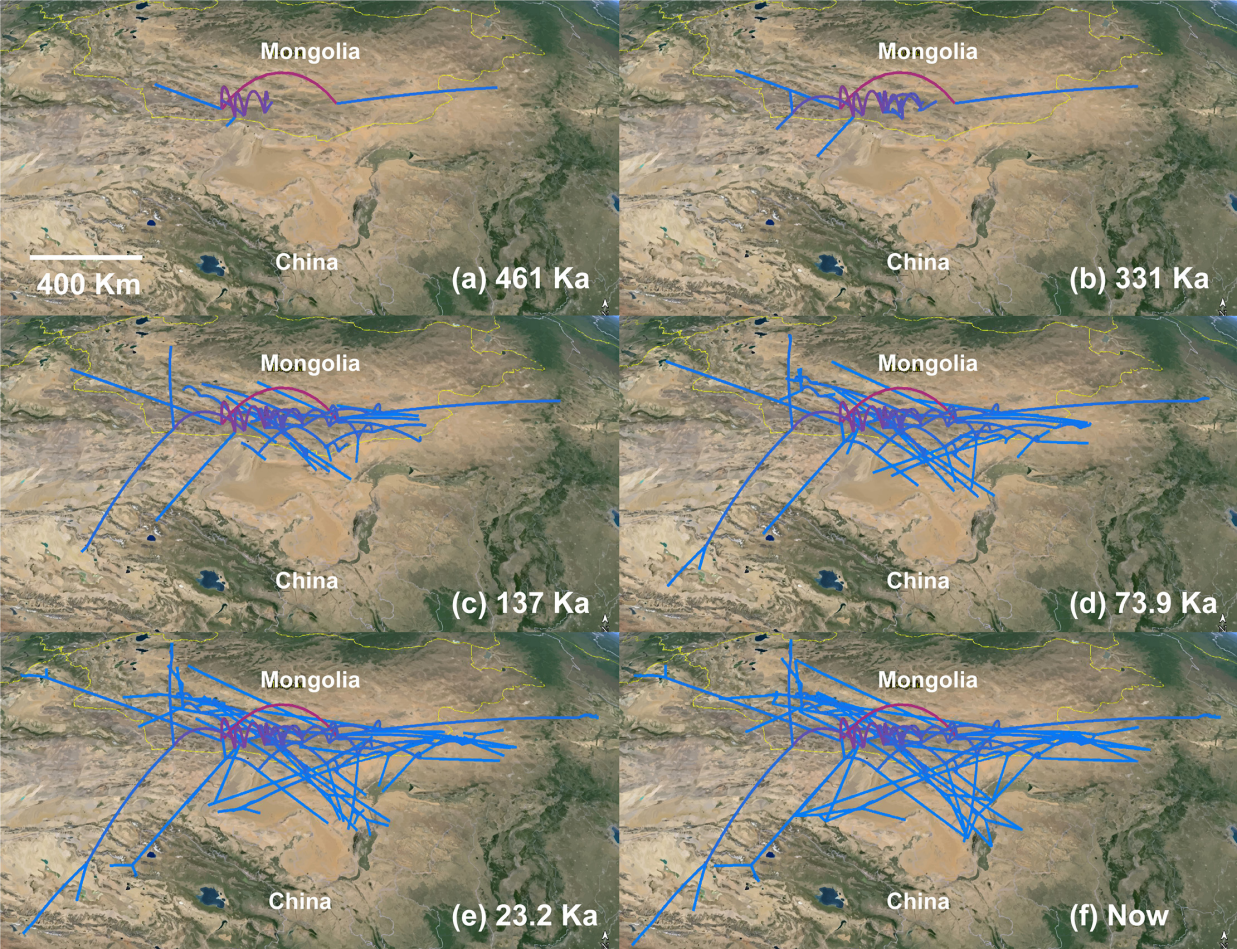
Results of the phylogeographic diffusion analysis. The time between 461 and 331ka refers to the interglacial period before the Penultimate Glaciation; 331–137 Ka corresponds to the Penultimate Glaciation; 137–73 Ka was the Last Interglacial and 23.2 Ka was the Last Glacial Maximum. The timeline was divided based on the chronology of the Quaternary glaciations in China summarized by [52].

The results of the phylogeographic analysis (Fig 6) showed that the range have widely expanded during the Penultimate Glaciation (MIS 6–10, 333–136 Ka) and the LIG (136–73 Ka). It was especially rapid during the LIG (only during 60 Ka). After the LGM (23 Ka) the range almost reached the current range and barely changed afterwards.

**Fig 7:**
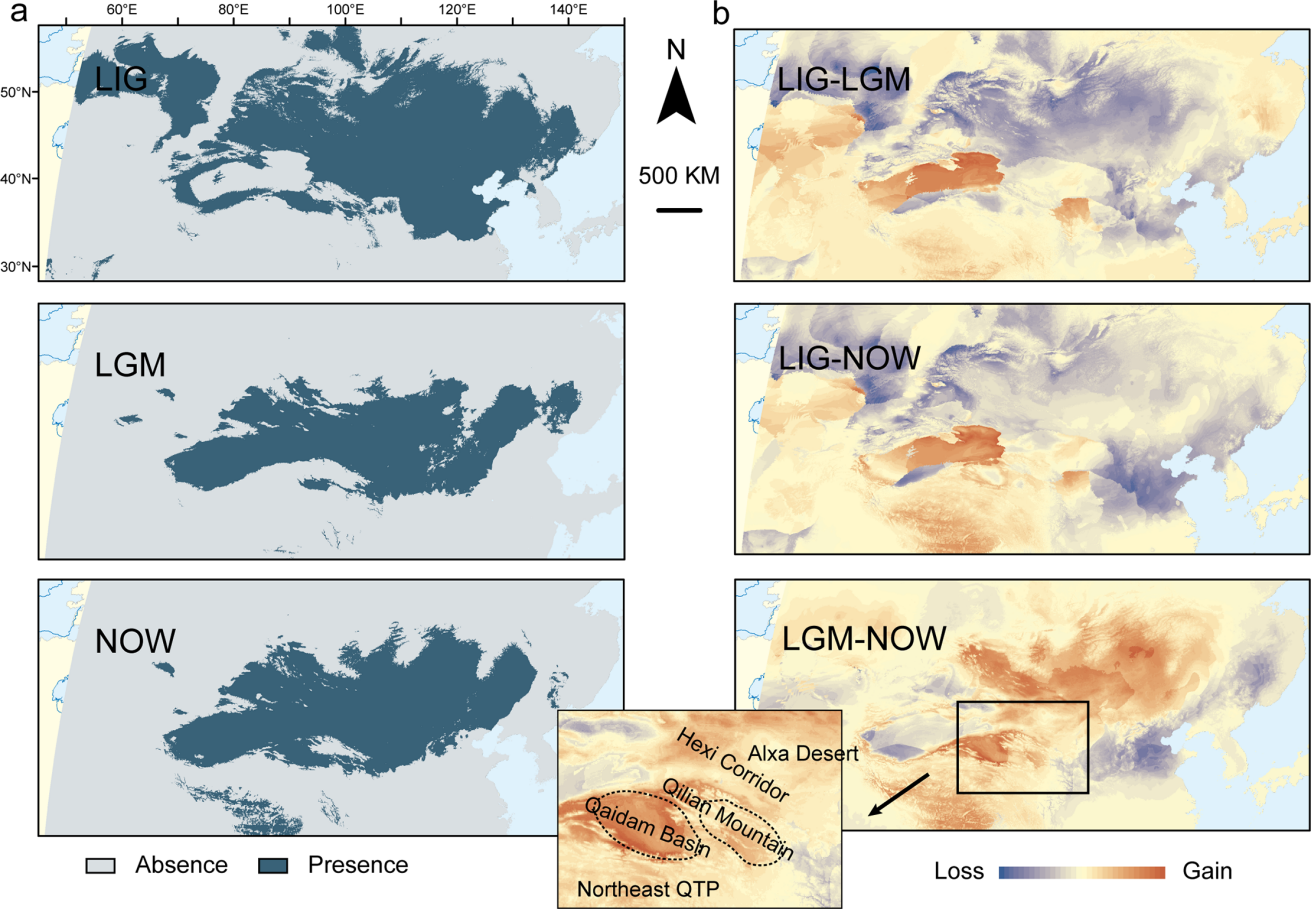
**(a) Ecological niche modelling (ENM) of suitable habitats for *P*. *roborovskii* generated by MaXent, and (b) differences in occurrence between the two periods, with losses (blue) and gains (red) of suitable habitats relative to the latter period**. The simulations were conducted based on the climatic condition of the Last Interglacial (LIG, first row), the Last Glacial Maximum (LGM, middle row) and the current climate (last row). The continental margin in the LGM differs from the others because of the lower sea level at the time. The suitable habitat was based on the 10% logistic training threshold estimated by Maxent. The geographical features discussed in the main text were labelled.

The ENM showed an excellent predictive power with relatively high AUC values (LGM, AUC value: 0.942; LIG, AUC value: 0.941). Furthermore, the tested data offered a significantly better prediction than that provided by the random model for all the 11 common thresholds. The binomial probabilities were significant (P = 0.008). The results of the ENM showed that the potential LIG distributions were the most widespread In contrast, the potential distribution of the LGM was greatly restricted, which shows more similarities to the current conditions (Fig 7A). Suitable habitats in the LGM showed a large contraction in the north and east relative to the LIG. Instead, the current suitable habitats expanded into the north and shifted towards the centre in comparison with the LGM (Fig 7B).

## Discussion

### Refugium in the Mongolia Plateau

Congruent results supported our MP refugium hypothesis that suggests that the MP served as a preservation centre for *P*. *roborovskii* during the unfavourable climatic conditions of the Quaternary. However, it was a glacial refugium instead of an interglacial refugium, as we expected (see discussion below). The genetic diversity in the localities of the MP was highest among all of the sampled localities, while continuous suitable habitat remained throughout the Quaternary glacial fluctuations due to the ENM.

One major phylogenetic discontinuity for *P*. *roborovskii* was the poorly resolved phylogenetic relationship of the Central clade. This clade showed no definite relationships among haplotypes and their geographical distributions, while samples collected from adjacent areas (e.g., Kazakhstan and Tuva) showed a genetic subdivision in different levels. There are no insurmountable barriers within this area. Furthermore, continuous suitable habitats revealed by the results of the ENM in the MP are suggestive of a high environmental homogeneity. Moreover, the MP, especially in its southern part, maintained suitable habitats for *P*. *roborovskii* throughout the harsh climatic fluctuations in the ecological niche modelling. Phylogeographic diffusion also showed a history of widespread gene flow and multiple colonization events within each locality of the MP. Therefore, integrated with evidence from the interpolation of genetic diversity and the results from phylogeographic analysis, the MP demonstrated to serve as origin, preservation and spread centre for *P*. *roborovskii*.

In contrast, the low genetic diversity found in other areas (adjacent areas) is indicative of a subsequent colonization [1], as supported by the temporal-spatial diffusion pattern. These areas possibly experienced strong bottleneck and selective sweep effects [53]. The star-shaped network also supported such scenario, which is similar to the pattern reported in the red-neck snow finch (*Pyrgilauda ruficollis*) [8] and two lizard species of the genus *Phrynocephalus* [54]. Moreover, the divergence pattern of the four localities around the Qaidam Basin is consistent with that of another sympatric species, the plateau zokor (*Eospalax baileyi*) [55]. The congruence of the geographical distributions of these species suggests that their genetic structures have been shaped in a similar way by the geographical isolation as a result of the Quaternary climatic oscillations. These localities were colonized with different ancestors and from different routes due to the phylogeographic diffusion.

### Glacial refugia and longitudinal shifts

With the integration of demographic and geographical analyses, our results showed a congruence pattern: *P*. *roborovskii* increased its range and population size during the interglacial periods instead of during the glacial periods. This outcome contradicts our hypothesis that *P*. *roborovskii* may have increased its demographic and geological range during the glacial periods based on its continent-adapted characters. Meanwhile, the diffusion process during the LIG also progressed much more rapidly than that during the glacial periods.

[5] reported a glacial expansion and an interglacial contraction of another continent-adapted species, the European ground squirrel (*Spermophilus citellus*, EGS). During the glacial periods, the EGS expanded further to the north and west as well as to a steppe habitat, while in the interglacial periods, the distribution contracted back to the Pannonian refugium. The discrepant pattern between these two species is potentially due to different precipitation conditions in the suitable habitats. Precipitation is thought to be the most important environmental correlate of species richness patterns in temperate and tropic regions, and the most fatal environmental factor for species adapted to arid environments [56]. Unlike the EGS, which dwells in short steppe grasslands with well-drained soils [57, 58], *P*. *roborovskii* is a sandy desert-dwelling species [57, 58]. Thus, the habitat of *P*. *roborovskii* may be more arid than that of the EGS. During the glacial periods, the reduction of the East Asian summer monsoon led to cooling temperatures and reduced rainfall all over China [59]. Steppe and even desert extended eastward to the modern coastline, which is now composed of temperate deciduous forests [60]. Therefore, the MP did not have glaciers [61], but it became more arid and colder than any other place in the same latitude [60]. Indeed, the annual precipitation of this region during the LGM was less than 100 mm [62]. Though most parts maintained suitable habitats, certain areas became uninhabitable for *P*. *roborovskii*. Based on the results of the ENM, the suitable habitats in the LGM showed a two-sided shrinkage in comparison with the current situation. The high potential distribution areas were shifted towards the east of the MP, which resulted in the Qaidam Basin, the Hexi Corridor and the Alxa Desert as less hospitable for *P*. *roborovskii*. Such scenario was also supported by palaeogeographic evidence. Due to the glaciation, the snowmelt in the Qilian Mountain was intercepted and that was the main water source for the northeast QTP and the Hexi Corridor [63].

In addition to the glacial arid tendency, another factor that probably worsened the conditions was the variation of vegetation. A study on the desert shrub *Nitraria sphaerocarpa*, which is important as forage for *P*. *roborovskii* [64], showed that this species reduced and fragmented its range during the LGM [12]. As a consequence, its habitat contracted during the glacial periods, in contrast with that of the European ground squirrels. As precipitation increased during the interglacial periods, the Hexi Corridor and the Qaidam Basin became hospitable again. The suitable habitats greatly expanded in several directions. The population growth took place within these interglacial periods. The importance of precipitation and other water resources for species adapted to arid environments was also characterized in the yarkand hare (*Lepus yarkandensis*) [65]. The biogeographical pattern of the yarkand hare showed an important effect of drainage in the southwest of the Tarim Basin to its refugia which was retained along the drainages during the glacial periods.

Nevertheless, a distinct longitudinal shift between the LGM and the current condition further indicated an important effect of the longitudinal variable on shaping the potential habitats, which partially supported our ROCG hypothesis. As the range expanded during the LIG, the longitudinal shifts was, therefore, not so distinct between glacial and interglacial periods. Moreover, the difference between the LIG and LGM suggests that the longitudinal shift between the LGM and the current condition is not simply due to the variation of the sea level. Though the lower sea level in the glacial period may results in a longitudinal variation, the eastern areas in the LIG still showed a higher suitability than the LGM, which suggests that the habitat actually shifted along the longitude from the LGM to the current condition.

Moreover, the low hospitality of the MP in the north edge during glacial periods might be the results of the low temperatures during the glacial periods. The temperatures are much lower in the north of the MP during both the present and the LGM conditions [66]. This shift pattern is similar to those that displayed a latitudinal shift during glacial fluctuations (e.g., American pika, [3]). Such pattern indicated that the latitudinal shift more likely reflect a distributional variation pattern along temperature gradients during climatic fluctuations, while the precipitation was represented by a longitudinal variable. Therefore, the range contraction of *P*. *roborovskii* during the glacial periods is not simply shaped by reduced precipitations alone but rather by both precipitation and temperature. Considering these results and the historical demography and phylogeographic diffusion, we were able to provide robust evidences that allowed us to reconstruct the reaction pattern for species adapted to arid environments during the glacial oscillations in the MP and, possibly, for Central Asia as a whole.

## Conclusion

In conclusion, the Mongolian Plateau played an important role in the evolutionary history of *P*. *roborovskii* during the Quaternary climate fluctuations, both as origin and preservation centre. Incontrast, other regions were either colonized or recolonized more recently. The low resolution in phylogenetic relationships further resulted from the homogeneous landscape and long-standing continuous suitable habitats in the MP throughout the Quaternary climatic fluctuations. The homogeneity provided opportunity for a wide gene flow. The glacial contraction and interglacial expansion showed different patterns compared with those of the European steppe species and highlighted the importance of precipitation in shaping the range of continent-adapted species in arid and semi-arid areas of Central and Northern Asia. Though longitudinal shifts only occurred between the LGM and the current conditions, the pattern further revealed the precipitation, which played an important role in shaping the distributions of species adapted to arid environments. Moreover, the range variation was potentially shaped by both temperature and precipitation, in the form of both latitudinal and longitudinal shifts during the climatic fluctuations. However, this pattern need to be verified with more species and with more specific studies about the effects of environmental factors on ecological and physiological characteristics.

## Supporting Information

S1 Fig Full interpolation of the genetic diversity. Red indicates a high genetic diversity, and blue indicates a low diversity. Dots represent sampling localities used to calculate the genetic diversity.
(TIF)

S1 Table Sample localities, references and GenBank accession numbers. (XLSX)
