# Supplementary figures and images for: Continental Refugium in the Mongolian Plateau during Quaternary Glacial Oscillations: Phylogeography and Niche Modelling of the Endemic Desert Hamster, Phodopus roborovskii
Source: PLoS One. 2016 Feb 3;11(2):e0148182. doi: 10.1371/journal.pone.0148182 (PMC4740458; doi:10.1371/journal.pone.0148182)

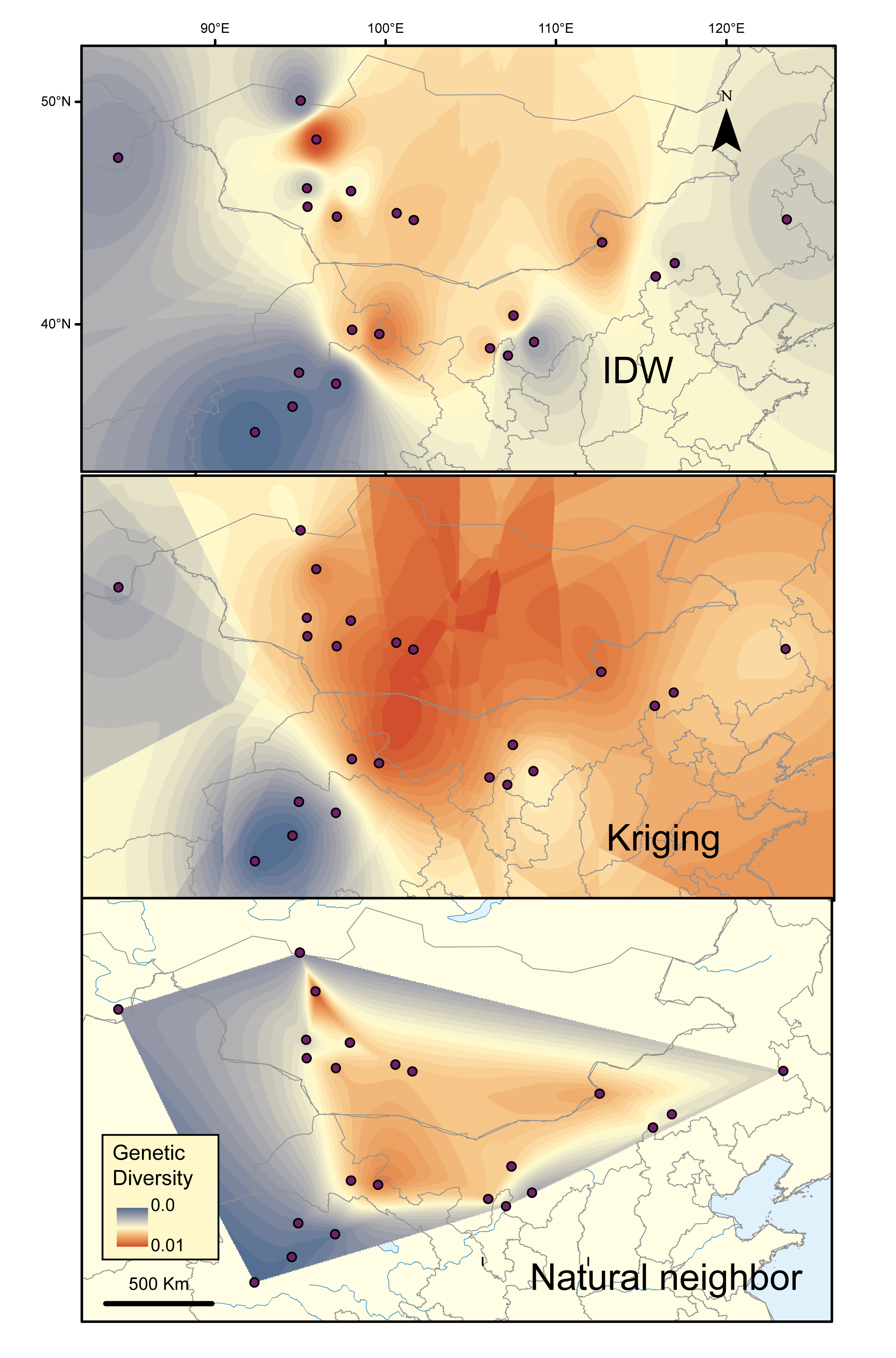

Supplement: S1 Fig — Red indicates a high genetic diversity, and blue indicates a low diversity. Dots represent sampling localities used to calculate the genetic diversity. (TIF) [file pone.0148182.s001.tif]
